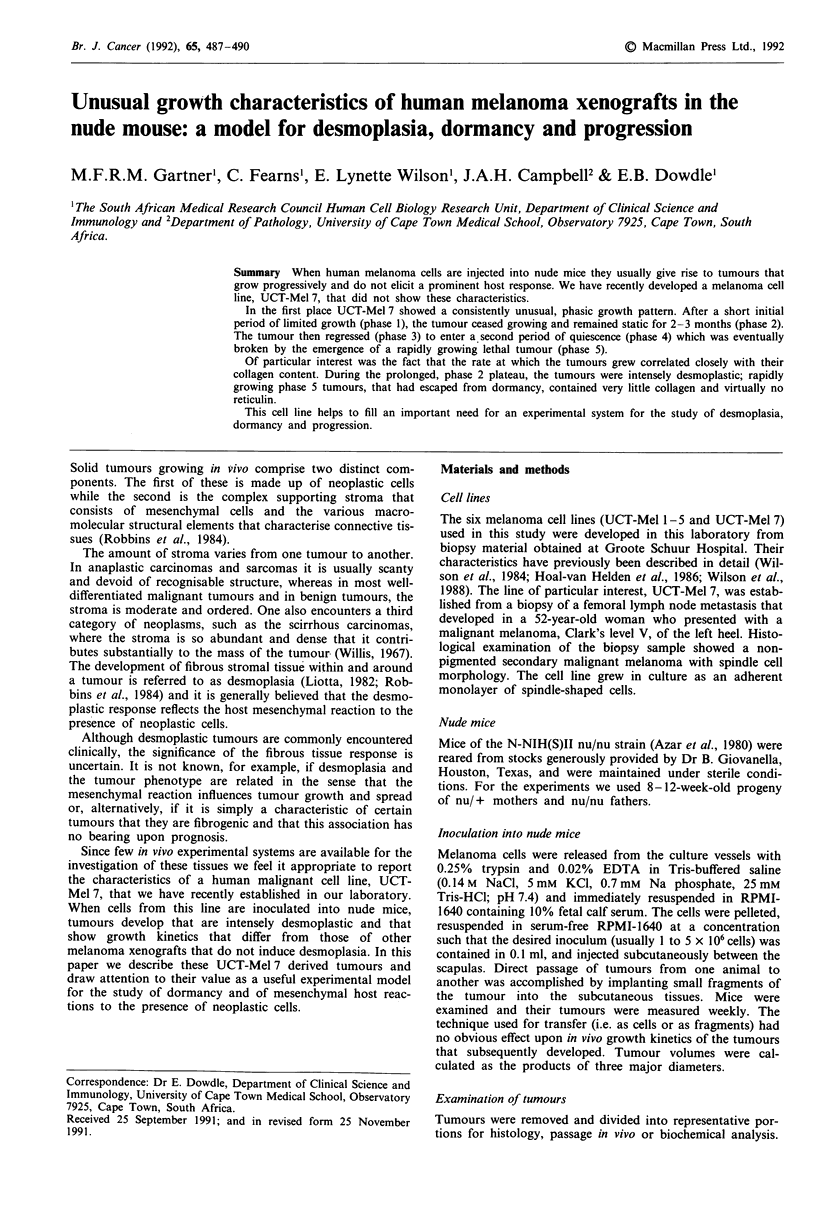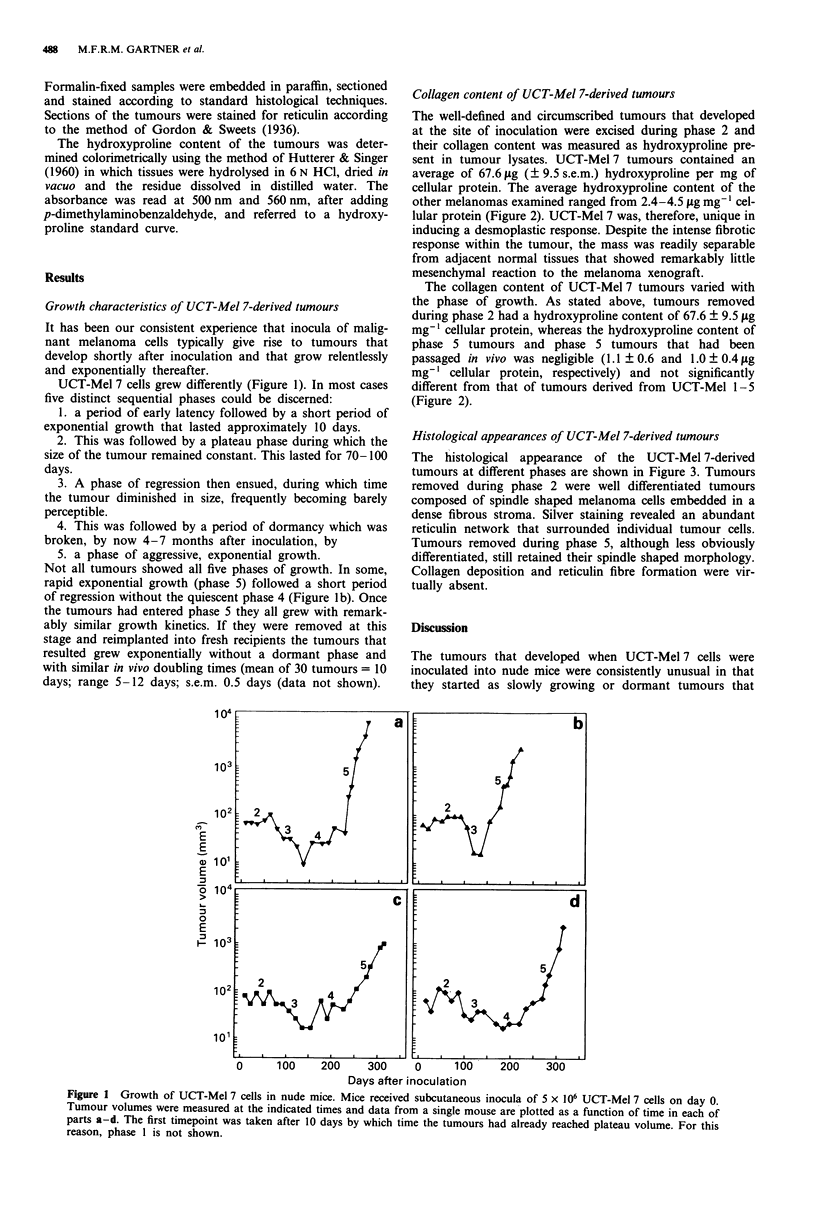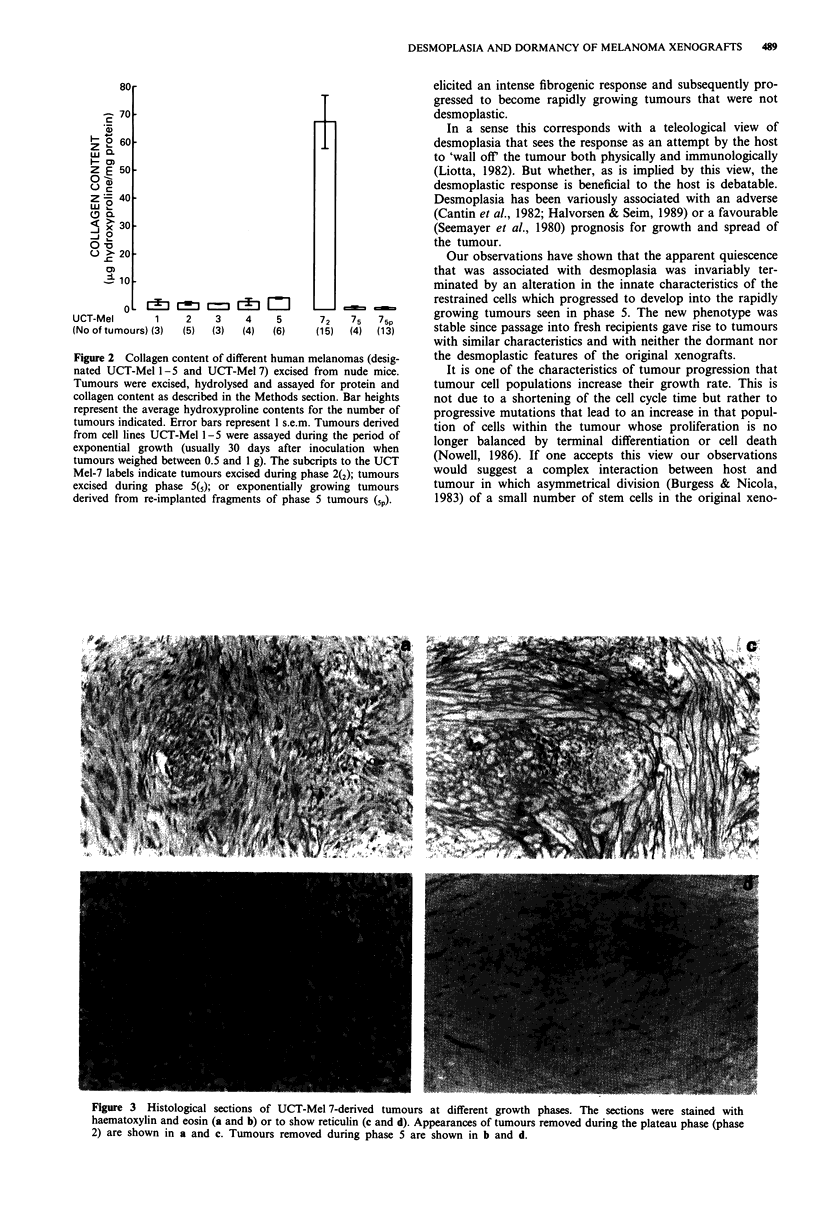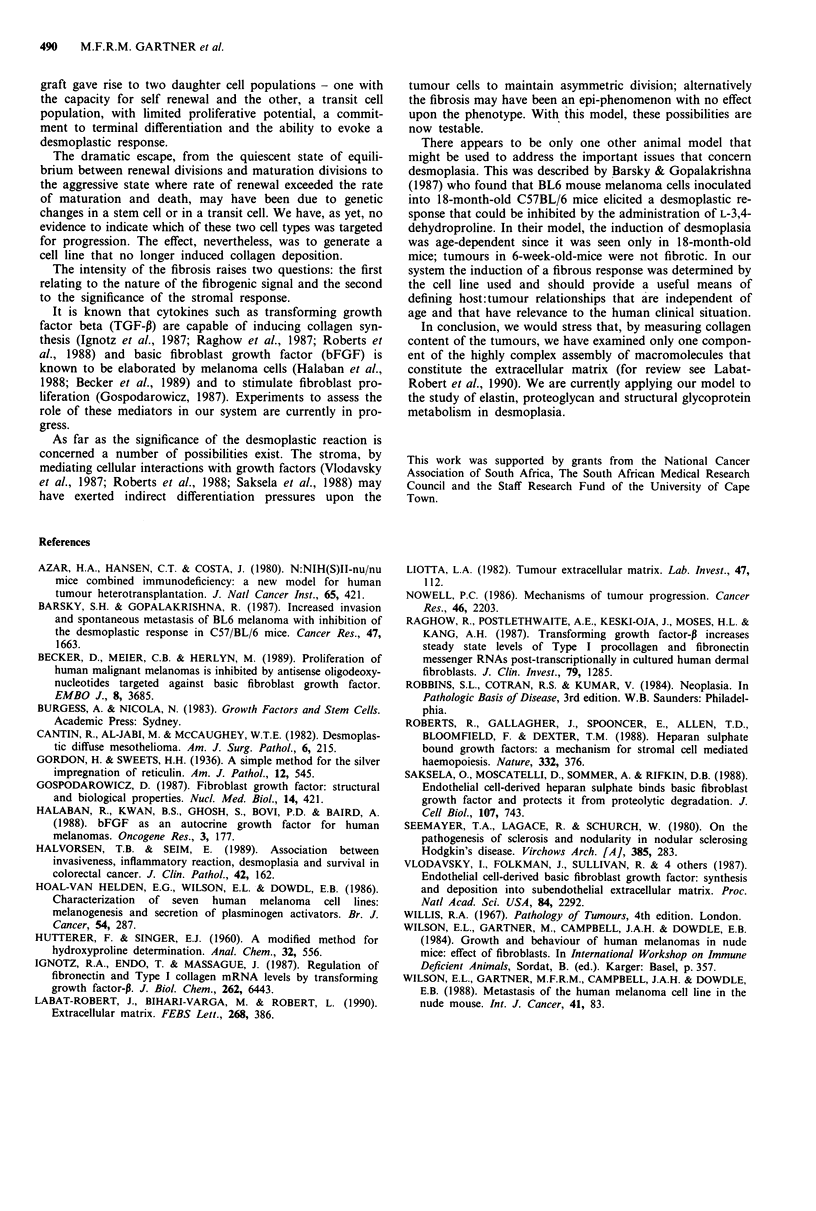# Unusual growth characteristics of human melanoma xenografts in the nude mouse: a model for desmoplasia, dormancy and progression.

**DOI:** 10.1038/bjc.1992.101

**Published:** 1992-04

**Authors:** M. F. Gartner, C. Fearns, E. L. Wilson, J. A. Campbell, E. B. Dowdle

**Affiliations:** Department of Clinical Science and Immunology, University of Cape Town Medical School, South Africa.

## Abstract

**Images:**


*Br. J. Cancer* (1992), **65**, 487–490 © Macmillan Press Ltd., 1992

### Unusual growth characteristics of human melanoma xenografts in the nude mouse: a model for desmoplasia, dormancy and progression

M.F.R.M. Gartner[1], C. Fearns[1], E. Lynette Wilson[1], J.A.H. Campbell[2] & E.B. Dowdle[1]

[1]*The South African Medical Research Council Human Cell Biology Research Unit, Department of Clinical Science and Immunology and* [2]*Department of Pathology, University of Cape Town Medical School, Observatory 7925, Cape Town, South Africa.*

**Summary** When human melanoma cells are injected into nude mice they usually give rise to tumours that grow progressively and do not elicit a prominent host response. We have recently developed a melanoma cell line, UCT-Mel 7, that did not show these characteristics.

In the first place UCT-Mel 7 showed a consistently unusual, phasic growth pattern. After a short initial period of limited growth (phase 1), the tumour ceased growing and remained static for 2–3 months (phase 2). The tumour then regressed (phase 3) to enter a second period of quiescence (phase 4) which was eventually broken by the emergence of a rapidly growing lethal tumour (phase 5).

Of particular interest was the fact that the rate at which the tumours grew correlated closely with their collagen content. During the prolonged, phase 2 plateau, the tumours were intensely desmoplastic; rapidly growing phase 5 tumours, that had escaped from dormancy, contained very little collagen and virtually no reticulin.

This cell line helps to fill an important need for an experimental system for the study of desmoplasia, dormancy and progression.

Solid tumours growing *in vivo* comprise two distinct components. The first of these is made up of neoplastic cells while the second is the complex supporting stroma that consists of mesenchymal cells and the various macromolecular structural elements that characterise connective tissues (Robbins *et al*., 1984).

The amount of stroma varies from one tumour to another. In anaplastic carcinomas and sarcomas it is usually scanty and devoid of recognisable structure, whereas in most well-differentiated malignant tumours and in benign tumours, the stroma is moderate and ordered. One also encounters a third category of neoplasms, such as the scirrhous carcinomas, where the stroma is so abundant and dense that it contributes substantially to the mass of the tumour (Willis, 1967). The development of fibrous stromal tissue within and around a tumour is referred to as desmoplasia (Liotta, 1982; Robbins *et al*., 1984) and it is generally believed that the desmoplastic response reflects the host mesenchymal reaction to the presence of neoplastic cells.

Although desmoplastic tumours are commonly encountered clinically, the significance of the fibrous tissue response is uncertain. It is not known, for example, if desmoplasia and the tumour phenotype are related in the sense that the mesenchymal reaction influences tumour growth and spread or, alternatively, if it is simply a characteristic of certain tumours that they are fibrogenic and that this association has no bearing upon prognosis.

Since few *in vivo* experimental systems are available for the investigation of these tissues we feel it appropriate to report the characteristics of a human malignant cell line, UCT-Mel 7, that we have recently established in our laboratory. When cells from this line are inoculated into nude mice, tumours develop that are intensely desmoplastic and that show growth kinetics that differ from those of other melanoma xenografts that do not induce desmoplasia. In this paper we describe these UCT-Mel 7 derived tumours and draw attention to their value as a useful experimental model for the study of dormancy and of mesenchymal host reactions to the presence of neoplastic cells.

Correspondence: Dr E. Dowdle, Department of Clinical Science and Immunology, University of Cape Town Medical School, Observatory 7925, Cape Town, South Africa.

Received 25 September 1991; and in revised form 25 November 1991.

### Materials and methods

### *Cell lines*

The six melanoma cell lines (UCT-Mel 1–5 and UCT-Mel 7) used in this study were developed in this laboratory from biopsy material obtained at Groote Schuur Hospital. Their characteristics have previously been described in detail (Wilson *et al*., 1984; Hoal-van Helden *et al*., 1986; Wilson *et al*., 1988). The line of particular interest, UCT-Mel 7, was established from a biopsy of a femoral lymph node metastasis that developed in a 52-year-old woman who presented with a malignant melanoma, Clark's level V, of the left heel. Histological examination of the biopsy sample showed a non-pigmented secondary malignant melanoma with spindle cell morphology. The cell line grew in culture as an adherent monolayer of spindle-shaped cells.

### *Nude mice*

Mice of the N-NIH(S)II nu/nu strain (Azar *et al*., 1980) were reared from stocks generously provided by Dr B. Giovanella, Houston, Texas, and were maintained under sterile conditions. For the experiments we used 8–12-week-old progeny of nu/+ mothers and nu/nu fathers.

### *Inoculation into nude mice*

Melanoma cells were released from the culture vessels with 0.25% trypsin and 0.02% EDTA in Tris-buffered saline (0.14 M NaCl, 5 mM KCl, 0.7 mM Na phosphate, 25 mM Tris-HCl; pH 7.4) and immediately resuspended in RPMI-1640 containing 10% fetal calf serum. The cells were pelleted, resuspended in serum-free RPMI-1640 at a concentration such that the desired inoculum (usually 1 to $5 \times 10^6$ cells) was contained in 0.1 ml, and injected subcutaneously between the scapulas. Direct passage of tumours from one animal to another was accomplished by implanting small fragments of the tumour into the subcutaneous tissues. Mice were examined and their tumours were measured weekly. The technique used for transfer (i.e. as cells or as fragments) had no obvious effect upon *in vivo* growth kinetics of the tumours that subsequently developed. Tumour volumes were calculated as the products of three major diameters.

### *Examination of tumours*

Tumours were removed and divided into representative portions for histology, passage *in vivo* or biochemical analysis.

Formalin-fixed samples were embedded in paraffin, sectioned and stained according to standard histological techniques. Sections of the tumours were stained for reticulin according to the method of Gordon & Sweets (1936).

The hydroxyproline content of the tumours was determined colorimetrically using the method of Hutterer & Singer (1960) in which tissues were hydrolysed in 6 N HCl, dried *in vacuo* and the residue dissolved in distilled water. The absorbance was read at 500 nm and 560 nm, after adding *p*-dimethylaminobenzaldehyde, and referred to a hydroxyproline standard curve.

### Results

### *Growth characteristics of UCT-Mel 7-derived tumours*

It has been our consistent experience that inocula of malignant melanoma cells typically give rise to tumours that develop shortly after inoculation and that grow relentlessly and exponentially thereafter.

UCT-Mel 7 cells grew differently (Figure 1). In most cases five distinct sequential phases could be discerned:

1. a period of early latency followed by a short period of exponential growth that lasted approximately 10 days.
2. This was followed by a plateau phase during which the size of the tumour remained constant. This lasted for 70–100 days.
3. A phase of regression then ensued, during which time the tumour diminished in size, frequently becoming barely perceptible.
4. This was followed by a period of dormancy which was broken, by now 4–7 months after inoculation, by
5. a phase of aggressive, exponential growth.

Not all tumours showed all five phases of growth. In some, rapid exponential growth (phase 5) followed a short period of regression without the quiescent phase 4 (Figure 1b). Once the tumours had entered phase 5 they all grew with remarkably similar growth kinetics. If they were removed at this stage and reimplanted into fresh recipients the tumours that resulted grew exponentially without a dormant phase and with similar *in vivo* doubling times (mean of 30 tumours = 10 days; range 5–12 days; s.e.m. 0.5 days (data not shown).

### *Collagen content of UCT-Mel 7-derived tumours*

The well-defined and circumscribed tumours that developed at the site of inoculation were excised during phase 2 and their collagen content was measured as hydroxyproline present in tumour lysates. UCT-Mel 7 tumours contained an average of 67.6 μg (± 9.5 s.e.m.) hydroxyproline per mg of cellular protein. The average hydroxyproline content of the other melanomas examined ranged from 2.4–4.5 μg $mg^{-1}$ cellular protein (Figure 2). UCT-Mel 7 was, therefore, unique in inducing a desmoplastic response. Despite the intense fibrotic response within the tumour, the mass was readily separable from adjacent normal tissues that showed remarkably little mesenchymal reaction to the melanoma xenograft.

The collagen content of UCT-Mel 7 tumours varied with the phase of growth. As stated above, tumours removed during phase 2 had a hydroxyproline content of 67.6 ± 9.5 μg $mg^{-1}$ cellular protein, whereas the hydroxyproline content of phase 5 tumours and phase 5 tumours that had been passaged *in vivo* was negligible (1.1 ± 0.6 and 1.0 ± 0.4 μg $mg^{-1}$ cellular protein, respectively) and not significantly different from that of tumours derived from UCT-Mel 1–5 (Figure 2).

### *Histological appearances of UCT-Mel 7-derived tumours*

The histological appearance of the UCT-Mel 7-derived tumours at different phases are shown in Figure 3. Tumours removed during phase 2 were well differentiated tumours composed of spindle shaped melanoma cells embedded in a dense fibrous stroma. Silver staining revealed an abundant reticulin network that surrounded individual tumour cells. Tumours removed during phase 5, although less obviously differentiated, still retained their spindle shaped morphology. Collagen deposition and reticulin fibre formation were virtually absent.

### Discussion

The tumours that developed when UCT-Mel 7 cells were inoculated into nude mice were consistently unusual in that they started as slowly growing or dormant tumours that

**Figure 1** Growth of UCT-Mel 7 cells in nude mice. Mice received subcutaneous inocula of 5 × $10^6$ UCT-Mel 7 cells on day 0. Tumour volumes were measured at the indicated times and data from a single mouse are plotted as a function of time in each of parts **a**–**d**. The first timepoint was taken after 10 days by which time the tumours had already reached plateau volume. For this reason, phase 1 is not shown.

**Figure 2** Collagen content of different human melanomas (designated UCT-Mel 1–5 and UCT-Mel 7) excised from nude mice. Tumours were excised, hydrolysed and assayed for protein and collagen content as described in the Methods section. Bar heights represent the average hydroxyproline contents for the number of tumours indicated. Error bars represent 1 s.e.m. Tumours derived from cell lines UCT-Mel 1–5 were assayed during the period of exponential growth (usually 30 days after inoculation when tumours weighed between 0.5 and 1 g). The subcripts to the UCT Mel-7 labels indicate tumours excised during phase $2(_2)$; tumours excised during phase $5(_5)$; or exponentially growing tumours derived from re-implanted fragments of phase 5 tumours $(_{5p})$.

elicited an intense fibrogenic response and subsequently progressed to become rapidly growing tumours that were not desmoplastic.

In a sense this corresponds with a teleological view of desmoplasia that sees the response as an attempt by the host to 'wall off' the tumour both physically and immunologically (Liotta, 1982). But whether, as is implied by this view, the desmoplastic response is beneficial to the host is debatable. Desmoplasia has been variously associated with an adverse (Cantin *et al.*, 1982; Halvorsen & Seim, 1989) or a favourable (Seemayer *et al.*, 1980) prognosis for growth and spread of the tumour.

Our observations have shown that the apparent quiescence that was associated with desmoplasia was invariably terminated by an alteration in the innate characteristics of the restrained cells which progressed to develop into the rapidly growing tumours seen in phase 5. The new phenotype was stable since passage into fresh recipients gave rise to tumours with similar characteristics and with neither the dormant nor the desmoplastic features of the original xenografts.

It is one of the characteristics of tumour progression that tumour cell populations increase their growth rate. This is not due to a shortening of the cell cycle time but rather to progressive mutations that lead to an increase in that popultion of cells within the tumour whose proliferation is no longer balanced by terminal differentiation or cell death (Nowell, 1986). If one accepts this view our observations would suggest a complex interaction between host and tumour in which asymmetrical division (Burgess & Nicola, 1983) of a small number of stem cells in the original xeno-

**Figure 3** Histological sections of UCT-Mel 7-derived tumours at different growth phases. The sections were stained with haematoxylin and eosin (**a** and **b**) or to show reticulin (**c** and **d**). Appearances of tumours removed during the plateau phase (phase 2) are shown in **a** and **c**. Tumours removed during phase 5 are shown in **b** and **d**.

graft gave rise to two daughter cell populations – one with the capacity for self renewal and the other, a transit cell population, with limited proliferative potential, a commitment to terminal differentiation and the ability to evoke a desmoplastic response.

The dramatic escape, from the quiescent state of equilibrium between renewal divisions and maturation divisions to the aggressive state where rate of renewal exceeded the rate of maturation and death, may have been due to genetic changes in a stem cell or in a transit cell. We have, as yet, no evidence to indicate which of these two cell types was targeted for progression. The effect, nevertheless, was to generate a cell line that no longer induced collagen deposition.

The intensity of the fibrosis raises two questions: the first relating to the nature of the fibrogenic signal and the second to the significance of the stromal response.

It is known that cytokines such as transforming growth factor beta (TGF-$\beta$) are capable of inducing collagen synthesis (Ignotz *et al.*, 1987; Raghow *et al.*, 1987; Roberts *et al.*, 1988) and basic fibroblast growth factor (bFGF) is known to be elaborated by melanoma cells (Halaban *et al.*, 1988; Becker *et al.*, 1989) and to stimulate fibroblast proliferation (Gospodarowicz, 1987). Experiments to assess the role of these mediators in our system are currently in progress.

As far as the significance of the desmoplastic reaction is concerned a number of possibilities exist. The stroma, by mediating cellular interactions with growth factors (Vlodavsky *et al.*, 1987; Roberts *et al.*, 1988; Saksela *et al.*, 1988) may have exerted indirect differentiation pressures upon the tumour cells to maintain asymmetric division; alternatively the fibrosis may have been an epi-phenomenon with no effect upon the phenotype. With this model, these possibilities are now testable.

There appears to be only one other animal model that might be used to address the important issues that concern desmoplasia. This was described by Barsky & Gopalakrishna (1987) who found that BL6 mouse melanoma cells inoculated into 18-month-old C57BL/6 mice elicited a desmoplastic response that could be inhibited by the administration of L-3,4-dehydroproline. In their model, the induction of desmoplasia was age-dependent since it was seen only in 18-month-old mice; tumours in 6-week-old-mice were not fibrotic. In our system the induction of a fibrous response was determined by the cell line used and should provide a useful means of defining host:tumour relationships that are independent of age and that have relevance to the human clinical situation.

In conclusion, we would stress that, by measuring collagen content of the tumours, we have examined only one component of the highly complex assembly of macromolecules that constitute the extracellular matrix (for review see Labat-Robert *et al.*, 1990). We are currently applying our model to the study of elastin, proteoglycan and structural glycoprotein metabolism in desmoplasia.

This work was supported by grants from the National Cancer Association of South Africa, The South African Medical Research Council and the Staff Research Fund of the University of Cape Town.

### References

AZAR, H.A., HANSEN, C.T. & COSTA, J. (1980). N:NIH(S)II-nu/nu mice combined immunodeficiency: a new model for human tumour heterotransplantation. *J. Natl Cancer Inst.*, **65**, 421.

BARSKY, S.H. & GOPALAKRISHNA, R. (1987). Increased invasion and spontaneous metastasis of BL6 melanoma with inhibition of the desmoplastic response in C57/BL/6 mice. *Cancer Res.*, **47**, 1663.

BECKER, D., MEIER, C.B. & HERLYN, M. (1989). Proliferation of human malignant melanomas is inhibited by antisense oligodeoxynucleotides targeted against basic fibroblast growth factor. *EMBO J.*, **8**, 3685.

BURGESS, A. & NICOLA, N. (1983). *Growth Factors and Stem Cells.* Academic Press: Sydney.

CANTIN, R., AL-JABI, M. & MCCAUGHEY, W.T.E. (1982). Desmoplastic diffuse mesothelioma. *Am. J. Surg. Pathol.*, **6**, 215.

GORDON, H. & SWEETS, H.H. (1936). A simple method for the silver impregnation of reticulin. *Am. J. Pathol.*, **12**, 545.

GOSPODAROWICZ, D. (1987). Fibroblast growth factor: structural and biological properties. *Nucl. Med. Biol.*, **14**, 421.

HALABAN, R., KWAN, B.S., GHOSH, S., BOVI, P.D. & BAIRD, A. (1988). bFGF as an autocrine growth factor for human melanomas. *Oncogene Res.*, **3**, 177.

HALVORSEN, T.B. & SEIM, E. (1989). Association between invasiveness, inflammatory reaction, desmoplasia and survival in colorectal cancer. *J. Clin. Pathol.*, **42**, 162.

HOAL-VAN HELDEN, E.G., WILSON, E.L. & DOWDL, E.B. (1986). Characterization of seven human melanoma cell lines: melanogenesis and secretion of plasminogen activators. *Br. J. Cancer*, **54**, 287.

HUTTERER, F. & SINGER, E.J. (1960). A modified method for hydroxyproline determination. *Anal. Chem.*, **32**, 556.

IGNOTZ, R.A., ENDO, T. & MASSAGUE, J. (1987). Regulation of fibronectin and Type I collagen mRNA levels by transforming growth factor-$\beta$. *J. Biol. Chem.*, **262**, 6443.

LABAT-ROBERT, J., BIHARI-VARGA, M. & ROBERT, L. (1990). Extracellular matrix. *FEBS Lett.*, **268**, 386.

LIOTTA, L.A. (1982). Tumour extracellular matrix. *Lab. Invest.*, **47**, 112.

NOWELL, P.C. (1986). Mechanisms of tumour progression. *Cancer Res.*, **46**, 2203.

RAGHOW, R., POSTLETHWAITE, A.E., KESKI-OJA, J., MOSES, H.L. & KANG, A.H. (1987). Transforming growth factor-$\beta$ increases steady state levels of Type I procollagen and fibronectin messenger RNAs post-transcriptionally in cultured human dermal fibroblasts. *J. Clin. Invest.*, **79**, 1285.

ROBBINS, S.L., COTRAN, R.S. & KUMAR, V. (1984). Neoplasia. In *Pathologic Basis of Disease*, 3rd edition. W.B. Saunders: Philadelphia.

ROBERTS, R., GALLAGHER, J., SPOONCER, E., ALLEN, T.D., BLOOMFIELD, F. & DEXTER, T.M. (1988). Heparan sulphate bound growth factors: a mechanism for stromal cell mediated haemopoiesis. *Nature*, **332**, 376.

SAKSELA, O., MOSCATELLI, D., SOMMER, A. & RIFKIN, D.B. (1988). Endothelial cell-derived heparan sulphate binds basic fibroblast growth factor and protects it from proteolytic degradation. *J. Cell Biol.*, **107**, 743.

SEEMAYER, T.A., LAGACE, R. & SCHURCH, W. (1980). On the pathogenesis of sclerosis and nodularity in nodular sclerosing Hodgkin's disease. *Virchows Arch. [A]*, **385**, 283.

VLODAVSKY, I., FOLKMAN, J., SULLIVAN, R. & 4 others (1987). Endothelial cell-derived basic fibroblast growth factor: synthesis and deposition into subendothelial extracellular matrix. *Proc. Natl Acad. Sci. USA*, **84**, 2292.

WILLIS, R.A. (1967). *Pathology of Tumours*, 4th edition. London.

WILSON, E.L., GARTNER, M., CAMPBELL, J.A.H. & DOWDLE, E.B. (1984). Growth and behaviour of human melanomas in nude mice: effect of fibroblasts. In *International Workshop on Immune Deficient Animals*, Sordat, B. (ed.). Karger: Basel, p. 357.

WILSON, E.L., GARTNER, M.F.R.M., CAMPBELL, J.A.H. & DOWDLE, E.B. (1988). Metastasis of the human melanoma cell line in the nude mouse. *Int. J. Cancer*, **41**, 83.